# Triglyceride-glucose index and the prognosis of patients with heart failure: A meta-analysis

**DOI:** 10.17305/bb.2024.10559

**Published:** 2024-07-07

**Authors:** Zhaoxia Yu, Wei Liu, Bo Li, Yutang Chen, Jian Li

**Affiliations:** 1 Department of Critical Care Medicine, The First Affiliated Hospital of Xinjiang Medical University, Urumqi, China

**Keywords:** Heart failure (HF), triglyceride-glucose index (TyGI), prognosis, mortality, meta-analysis

## Abstract

The triglyceride-glucose index (TyGI) is a novel indicator of insulin resistance (IR), which has been associated with an increased risk of cardiovascular diseases (CVDs). The aim of this meta-analysis was to determine the association between TyGI and the prognosis of patients with heart failure (HF). Cohort studies relevant to the aim of the meta-analysis were retrieved by searching electronic databases, including PubMed, Web of Science, and Embase. A random-effects model was used to combine the data, incorporating the influence of between-study heterogeneity. Twelve studies involving 20,639 patients with HF were included. Pooled results showed that compared to patients with the lowest category of TyGI at baseline, those with the highest TyGI index were associated with a higher risk of all-cause mortality during follow-up (relative risk [RR] 1.71, 95% confidence interval [CI] 1.46–2.00; *P* < 0.001; *I*^2^ ═ 55%). Sensitivity analyses limited to studies after adjustment for confounding factors showed similar results (RR 1.89, 95% CI 1.67–2.21; *P* < 0.001; *I*^2^ ═ 13%). Subsequent meta-analyses also showed that a high TyGI at baseline was related to the incidence of cardiovascular (CV) death (RR 1.87, 95% CI 1.42–2.47; *P* < 0.001; *I*^2^ ═ 57%), HF rehospitalization (RR 1.33, 95% CI 1.04–1.69; *P* < 0.02; *I*^2^ ═ 46%), and major adverse CV events (RR 1.69, 95% CI 1.39–2.06; *P* < 0.001; *I*^2^ ═ 17%) during follow-up. In conclusion, a high TyGI may be associated with a poor clinical prognosis for patients with HF.

## Introduction

Heart failure (HF) represents the advanced and terminal stage of various cardiovascular diseases (CVDs) [1–3]. From a pathophysiological perspective, HF is characterized by an intrinsic deficiency in either the contraction or relaxation of the myocardium, leading to the activation of neurohormonal systems. This ultimately results in progressive deterioration of cardiac function and inadequate circulation to peripheral tissues [4]. With global aging and advancements in CVD treatment strategies, it is anticipated that the number of HF patients will continue to rise in the coming decades [5, 6]. Despite recent therapeutic developments for HF, the prognosis remains unfavorable for affected individuals [7]. Consequently, there is a crucial need to identify novel prognostic indicators for patients with HF.

Insulin resistance (IR) has been linked to the onset and progression of HF through its promotion of low-grade systemic inflammation, oxidative stress, and endothelial dysfunction [8]. Recent studies have proposed that the triglyceride-glucose index (TyGI), a metric derived from fasting plasma glucose (FPG) and triglyceride (TG) levels, can serve as an effective indicator of IR [9]. The TyGI has demonstrated a strong correlation with hyperinsulinemic-euglycemic clamp results, which are considered the gold standard for assessing IR [10–12]. Furthermore, mounting evidence suggests that a high TyGI is associated with an increased risk of CVD, including HF, in the general population [13–15]. However, prior research examining the relationship between TyGI and the prognosis of HF patients has produced inconclusive findings [16–27]. To address this gap, we conducted a meta-analysis to investigate the association between TyGI and the prognosis of patients with HF.

## Materials and methods

The Preferred Reporting Items for Systematic Reviews and Meta-Analyses (PRISMA) statement (2020) [28, 29] was followed in this study. The Cochrane Handbook [30] for systematic review and meta-analysis was referenced throughout the study.

### Literature analysis

Three major electronic databases—PubMed, Web of Science, and Embase—were used for the literature search, with predefined combined search terms including: (1) “TyG index” OR “triglyceride-glucose index” OR “triglyceride and glucose index” OR “triglyceride glucose index” OR “triacylglycerol glucose index” OR “TyGI,” combined with (2) “heart failure” OR “cardiac failure” OR “cardiac dysfunction.” Only studies involving human subjects and published in English were included. A second-round reference check of relevant articles was also conducted. The final database search was completed on January 12, 2024.

### Inclusion and exclusion criteria

The inclusion criteria were based on the PICOS principle:

(1) P (Patients): Individuals with a confirmed diagnosis of HF.
(2) I (Intervention): TyGI was measured at baseline according to the formula ln [TG (mg/dl) × FPG (mg/dl)]/2, with a high TyGI at baseline considered the exposure. The cutoff for defining a high TyGI was consistent with values used in the original studies. Baseline TyGI refers to the TyGI measured at admission for patients with hospitalized HF (typically acute HF [AHF]) and TyGI measured at enrollment for stable HF patients (typically chronic HF [CHF]).
(3) C (Comparison): Patients with a low baseline TyGI were considered the control group.
(4) O (Outcome): The primary outcome was the incidence of all-cause mortality during follow-up, compared between HF patients with the highest vs the lowest TyGI category at baseline. Secondary outcomes included the incidence of cardiovascular (CV) death, HF-related rehospitalization, and the composite outcome of major adverse CV events (MACE).
(5) S (Study design): Cohort studies, including both prospective and retrospective designs.

We excluded reviews, meta-analyses, studies analyzing TyGI as a continuous variable only, or studies lacking outcomes relevant to this meta-analysis, such as all-cause mortality, CV death, HF rehospitalization, and MACE. In cases of potential overlap in patient populations across multiple studies, only the study with the largest sample size was included.

### Data collection and quality assessment

Two independent authors conducted a thorough search of academic literature, performed data collection and analysis, and assessed the quality of the studies. Any discrepancies were resolved through discussion with the corresponding author to reach a final decision. Data on study details, design, patient diagnoses, sample size, age, sex, diabetic status, TyGI cutoffs, follow-up duration, reported outcomes, and variables adjusted in the regression models assessing the association between TyGI and clinical outcomes in HF patients were gathered. Study quality was assessed using the Newcastle–Ottawa Scale (NOS) [31], which involves scoring based on criteria, such as participant selection, group comparability, and outcome validity. The scale rates studies from 1 to 9 stars, with higher stars indicating better quality.

### Ethical statement

Ethical approval was not required for this study in accordance with local/national guidelines. Written informed consent to participate was also not required under local/national regulations.

### Statistical analysis

The association between TyGI and clinical outcomes in HF patients was presented using relative risk (RR) and corresponding 95% confidence intervals (CIs), comparing patients with the highest vs the lowest baseline TyGI. RRs and standard errors were calculated from the 95% CIs or *P* values, followed by logarithmic transformation to stabilize variance and normalize distribution [30]. Heterogeneity among studies was assessed using the Cochrane *Q* test and *I^2^* statistic [32, 33], with *I*^2^ > 50% indicating significant heterogeneity. A random-effects model was used for result aggregation considering the influence of clinical heterogeneity among the included studies [30], such as differences in HF type (acute/chronic), TyGI cutoffs, and follow-up duration. For the primary outcome of all-cause mortality, sensitivity analysis was limited to studies with multivariate analyses that adjusted for potential confounding factors. Multiple subgroup analyses were conducted to evaluate the influence of study characteristics, including HF type (acute vs chronic), reduced or preserved ejection fraction (HFrEF vs HFpEF), diabetes status, TyGI cutoffs, follow-up duration, and NOS ratings on the results. Medians of continuous variables were used to define subgroup cutoffs. For characteristics presented as continuous variables, such as the sample size, mean age, proportion of male or diabetic patients, and follow-up duration, a univariate meta-regression analysis was performed [30]. Publication bias was assessed through visual inspection of funnel plots for symmetry, followed by Egger’s regression test [34], where *P* < 0.05 indicated statistical significance. All analyses were conducted using RevMan Version 5.1 (Cochrane Collaboration, Oxford, UK) and Stata software version 12 (Stata Corporation, College Station, TX, USA).

## Results

### Study inclusion

**Figure 1.**
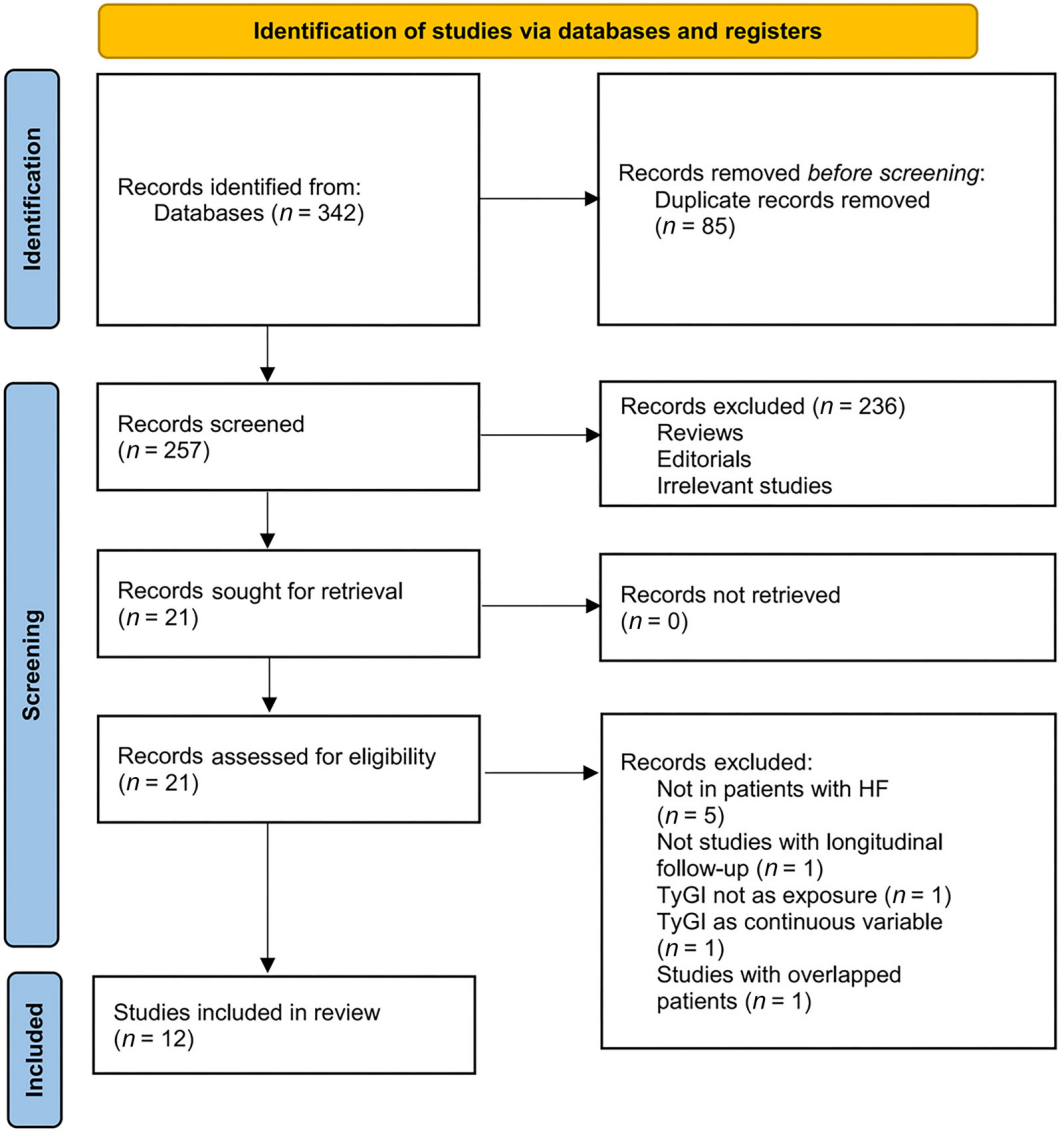
**Process of conducting literature search and identifying studies**. TyGI: Triglyceride-glucose index; HF: Heart failure.

The process of selecting relevant studies for inclusion in the meta-analysis is depicted in Figure 1. Initially, 342 potentially pertinent records were identified through comprehensive searches of three databases. Of these, 85 were excluded due to duplication. Subsequent screening of titles and abstracts led to the exclusion of an additional 236 studies that did not align with the objectives of the meta-analysis. The full texts of the remaining 21 records were independently reviewed by two authors, resulting in the removal of nine studies for various reasons detailed in Figure 1. Ultimately, 12 cohort studies were included [16–27] and deemed suitable for quantitative analysis.

### Overview of the studies’ characteristics

Table 1 provides a summary of the characteristics of the included studies. In total, one prospective cohort study [25] and 11 retrospective cohort studies [16–24, 26, 27] were included in the meta-analysis. These studies, published between 2021 and 2024, were performed in China, Portugal, Turkey, Japan, and the United States. All studies involved adult populations with HF, with patients’ mean ages ranging from 60.3 to 81.0 years. The methods for defining the cutoff for the TyGI varied among studies, using the median [16, 27], tertiles [17–19, 22, 25, 26], and quartiles [20, 21, 23, 24] of TyGI in the respective patient populations. The cutoff value for a high TyGI ranged from 8.65 to 13.2. Follow-up durations varied from within hospitalization to 60 months. The primary outcome, all-cause mortality, was reported in 11 studies [17–27], while secondary outcomes such as CV death, HF rehospitalization, and MACE were reported in four [17, 19, 25, 26], three [17, 20, 25], and three studies [19, 21, 23], respectively. Univariate analyses were used in three studies to report the association between TyGI and clinical outcomes in HF patients [16, 21, 24], while multivariate analyses were employed in the remaining nine studies [17–20, 22, 23, 25–27]. Variables, such as age, sex, hemodynamic parameters, comorbidities, ejection fraction, and concurrent medications were adjusted to varying extents across the studies. The NOS scores for the included studies ranged from 6 to 9 stars, indicating overall moderate to good quality (Table 2).

**Table 1:** Characteristics of the included studies

**Study**	**Country**	**Study design**	**Diagnosis**	**No. of patients**	**Mean age (years)**	**Men (%)**	**DM (%)**	**Methods to determine TyGI cutoff**	**Mean BMI (kg/m^2^)**	**Cutoff value for high TyGI**	**Follow-up duration (months)**	**Outcomes reported**	**Variables adjusted or matched**
Guo et al., 2021 [17]	China	RC	CHF	546	65.2	66.3	100	T3:T1	21.1	9.06	27.6	CV death; HF rehospitalization	Age, sex, BMI, SBP, DBP, HR, CRP, eGFR, NT-proBNP, HbA1c, LVEF, AF, NYHA class, and concurrent medications
Cunha et al., 2021 [16]	Portugal	RC	CHF	275	69	70.5	NR	Median	NR	8.65	60	All-cause mortality	None
Huang et al., 2022 [19]	China	RC	ADHF	932	61.8	62.1	32.8	T3:T1	24.2	9.32	15.7	All-cause mortality; CV death; MACE	Age, sex, BMI, SBP, DBP, HR, CRP, eGFR, BNP, HbA1c, SUA, LVEF, DM, HTN, VHD, AF, and concurrent medications
Han et al., 2022 [18]	China	RC	ADHF	4441	70.6	48.4	32.1	T3:T1	NR	8.78	During hospitalization	All-cause mortality	Age, sex, NYHA class, HR, SBP, albumin, TBIL, LDL-C, BUN, SCr, SUA, HGB, serum sodium, cTnI, NT-proBNP, LVEF, and the history of CAD, HTN, AF, DM, smoking, and concurrent medications
Shi et al., 2022 [20]	China	RC	HF	901	NR	44.5	25.8	Q4:Q1	20.9	NR	6	All-cause mortality; HF rehospitalization	Age, sex, BMI, NYHA class, CHF, hypertension, DM, CKD, LVEF, eGFR, hsCRP, BNP, albumin, cholesterol, and LDL-C, and concurrent medications
Ozcan et al., 2023 [22]	Turkey	RC	HFrEF after ICD	773	61.5	81.9	35.6	T3:T1	NR	13.2	38	All-cause mortality	Age, sex, HTN, DM, NYHA class, BUN, and LVEF
Zhou et al., 2023 [25]	China	PC	AHF, HFpEF	823	73	48.1	42	T3:T1	25.5	8.98	37.9	All-cause mortality; CV death; HF rehospitalization	Age, sex, BMI, SBP, HbA1c, HDL-C, SCr, Hb, cTnT, NT-proBNP, DM, hyperlipidemia, AF, LVEF, LAD, and concurrent medications
Zhou et al., 2023 [26]	China	RC	CHF	6697	64	68.4	44.6	T3:T1	25.2	8.93	46.8	All-cause mortality; CV death	Age, sex, BMI, smoking, drinking status, HbA1c, TBil, albumin, eGFR, TC, LDL-C, cTnT, sodium, NT-proBNP, LVEF and NYHA classification, HTN, DM, AF, previous MI, stroke, and concurrent medications
Yang et al., 2023 [24]	USA	RC	ADHF	1393	71	59	37.6	Q4:Q1	NR	NR	During hospitalization	All-cause mortality	None
Iwakura et al., 2023 [21]	Japan	RC	AHF, HFpEF	917	81	44.7	39.3	Q4:Q1	NR	NR	12.7	All-cause mortality; MACE	None
Sun et al., 2023 [23]	China	RC	Ischemic HF after PCI	2055	60.3	82.2	38.5	Q4:Q1	25.9	9.41	36	All-cause mortality; MACE	Age, sex, HR, BMI, NYHA class, prior PCI, platelet, albumin, TC, LDL-C, potassium, SUA, LVEF, coronary lesion severity, and concurrent medications
Cheng et al., 2024 [27]	China	RC	ADFH	886	71	55.5	0	Median	NR	9.44	During hospitalization	All-cause mortality	Age, sex, cardiogenic shock, NT-proBNP, albumin, TC, LDL-C, TBIL, comorbidities of HTN, CHD, AF, and concurrent medications

DM: Diabetes mellitus; TyGI: Triglyceride-glucose index; RC: Retrospective cohort; PC: Prospective cohort; CHF: Chronic heart failure; AHF: Acute heart failure; ADHF: Acute decompensated heart failure; HFrEF: Heart failure with reduced ejection fraction; HFpEF: Heart failure with preserved ejection fraction; ICD: Implantable cardioverter defibrillator; PCI: Percutaneous coronary intervention; NR: Not reported; T: Tertile; Q: Quartile; CV: Cardiovascular; HF: Heart failure; MACE: Major adverse cardiovascular events; BMI: Body mass index; SBP: Systolic blood pressure; DBP: Diastolic blood pressure; HR: Heart rate; CRP: C-reactive protein; eGFR: Estimated glomerular filtrating rate; NT-proBNP: N-terminal pro B-type natriuretic peptide; HbA1c: Hemoglobin A1c; LVEF: Left ventricular ejection fraction; AF: Atrial fibrillation; NYHA: New York Heart Association; BNP: B-type natriuretic peptide; SUA: Serum uric acids; HGB: Hemoglobin; DM: Diabetes mellitus; HTN: Hypertension; VHD: Valvular heart disease; TBIL: Total bilirubin; LDL-C: Low-density lipoprotein cholesterol; TC: Total cholesterol; MI: Myocardial infarction; BUN: Blood urea nitrogen; CAD: Coronary artery disease; CKD: Chronic kidney disease; cTnI: Cardiac troponin I; cTnT: Cardiac troponin T.

**Table 2:** Study quality evaluation via the Newcastle-Ottawa scale

**Study**	**Representativeness of the exposed cohort**	**Selection of the non-exposed cohort**	**Ascertainment of exposure**	**Outcome not present at baseline**	**Control for age and sex**	**Control for other confounding factors**	**Assessment of outcome**	**Enough long follow-up duration**	**Adequacy of follow-up of cohorts**	**Total**
Guo et al., 2021 [17]	0	1	1	1	1	1	1	1	1	8
Cunha et al., 2021 [16]	0	1	1	1	0	0	1	1	1	6
Huang et al., 2022 [19]	0	1	1	1	1	1	1	1	1	8
Han et al., 2022 [18]	1	1	1	1	1	1	1	0	1	8
Shi et al., 2022 [20]	0	1	1	1	1	1	1	0	1	7
Ozcan et al., 2023 [22]	0	1	1	1	1	1	1	1	1	8
Zhou et al., 2023 [25]	1	1	1	1	1	1	1	1	1	9
Zhou et al., 2023 [26]	1	1	1	1	1	1	1	1	1	9
Yang et al., 2023 [24]	1	1	1	1	0	0	1	0	1	6
Iwakura et al., 2023 [21]	0	1	1	1	0	0	1	1	1	6
Sun et al., 2023 [23]	0	1	1	1	1	1	1	1	1	8
Cheng et al., 2024 [27]	0	1	1	1	1	1	1	0	1	7

### Meta-analysis for the association between TyGI and all-cause mortality

**Figure 2.**
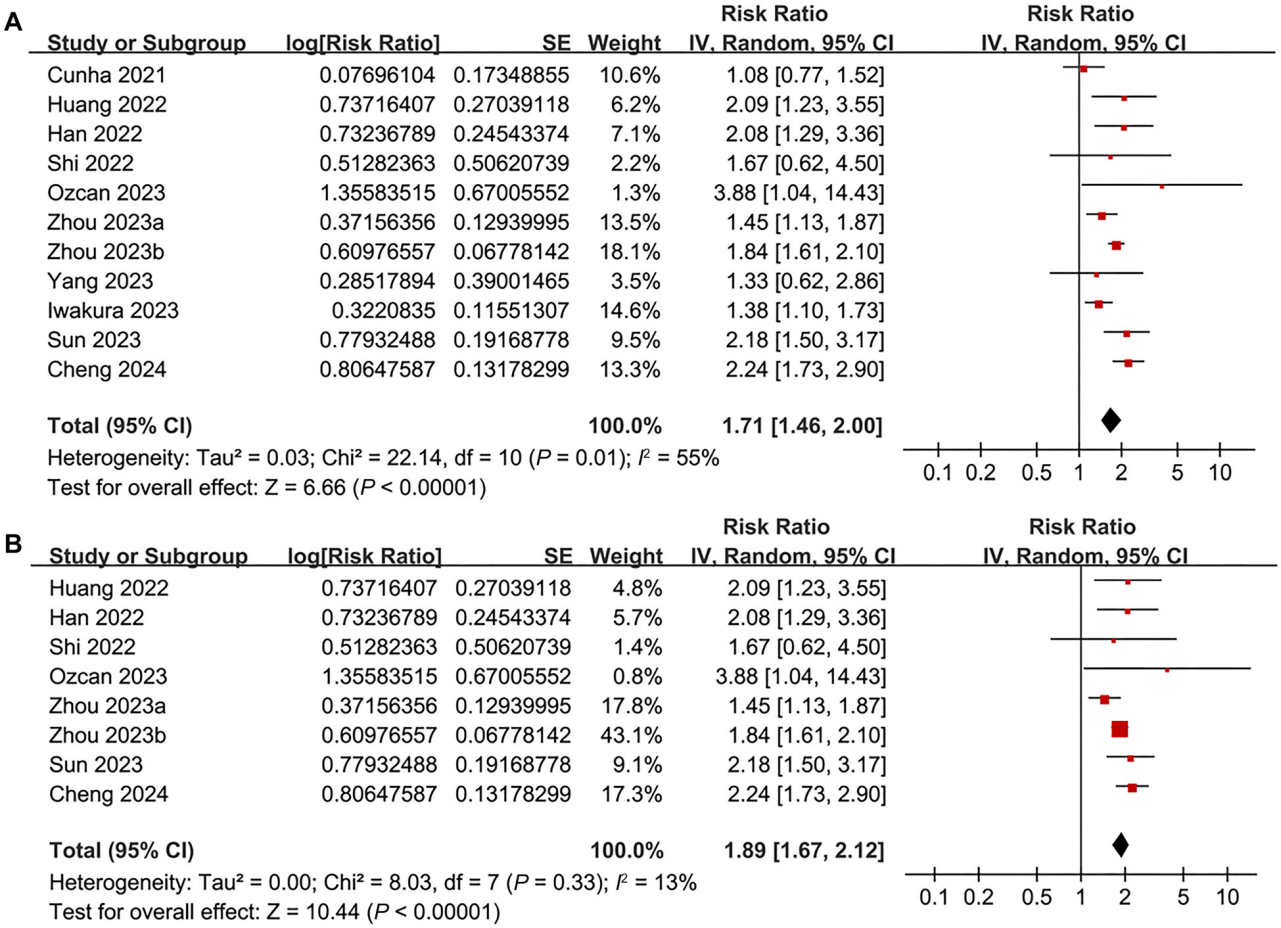
**Forest plots for the meta-analysis of the association between TyGI and all-cause mortality in patients with HF.** (A) Forest plot for the overall meta-analysis; (B) Forest plot for the sensitivity analysis limited to studies with adjustment for confounding factors. TyGI: Triglyceride-glucose index; HF: Heart failure; CI: Confidence interval.

**Figure 3.**
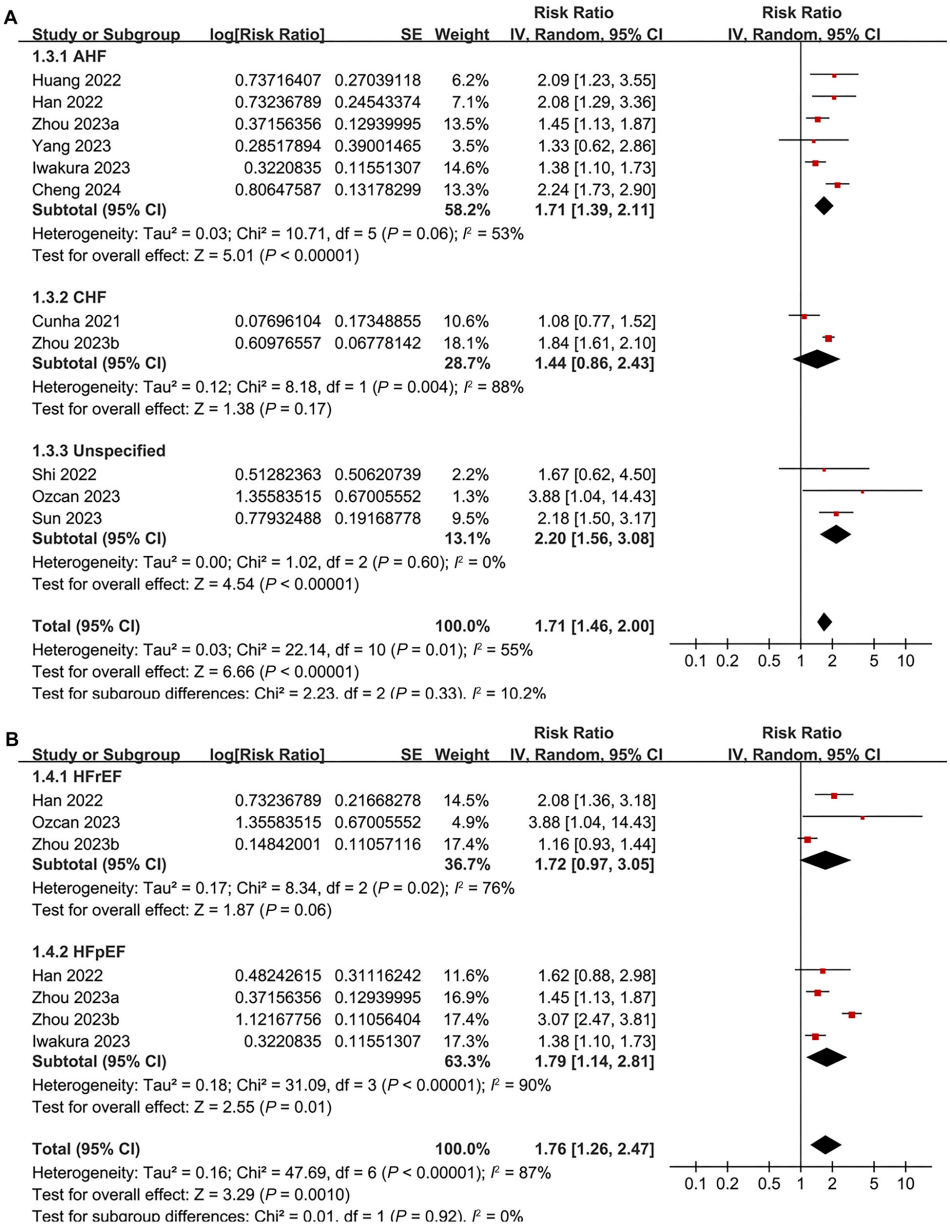
**Forest plots for the subgroup analyses of the association between TyGI and all-cause mortality of patients with HF.** (A) Forest plot for the subgroup analysis of acute versus chronic HF; (B) Forest plot for the subgroup analysis of HFrEF versus HFpEF. HF: Heart failure; AHF: Acute heart failure; CHF: Chronic heart failure; HFrEF: Heart failure with reduced ejection fraction; HFpEF: Heart failure preserved ejection fraction; CI: Confidence interval.

**Figure 4.**
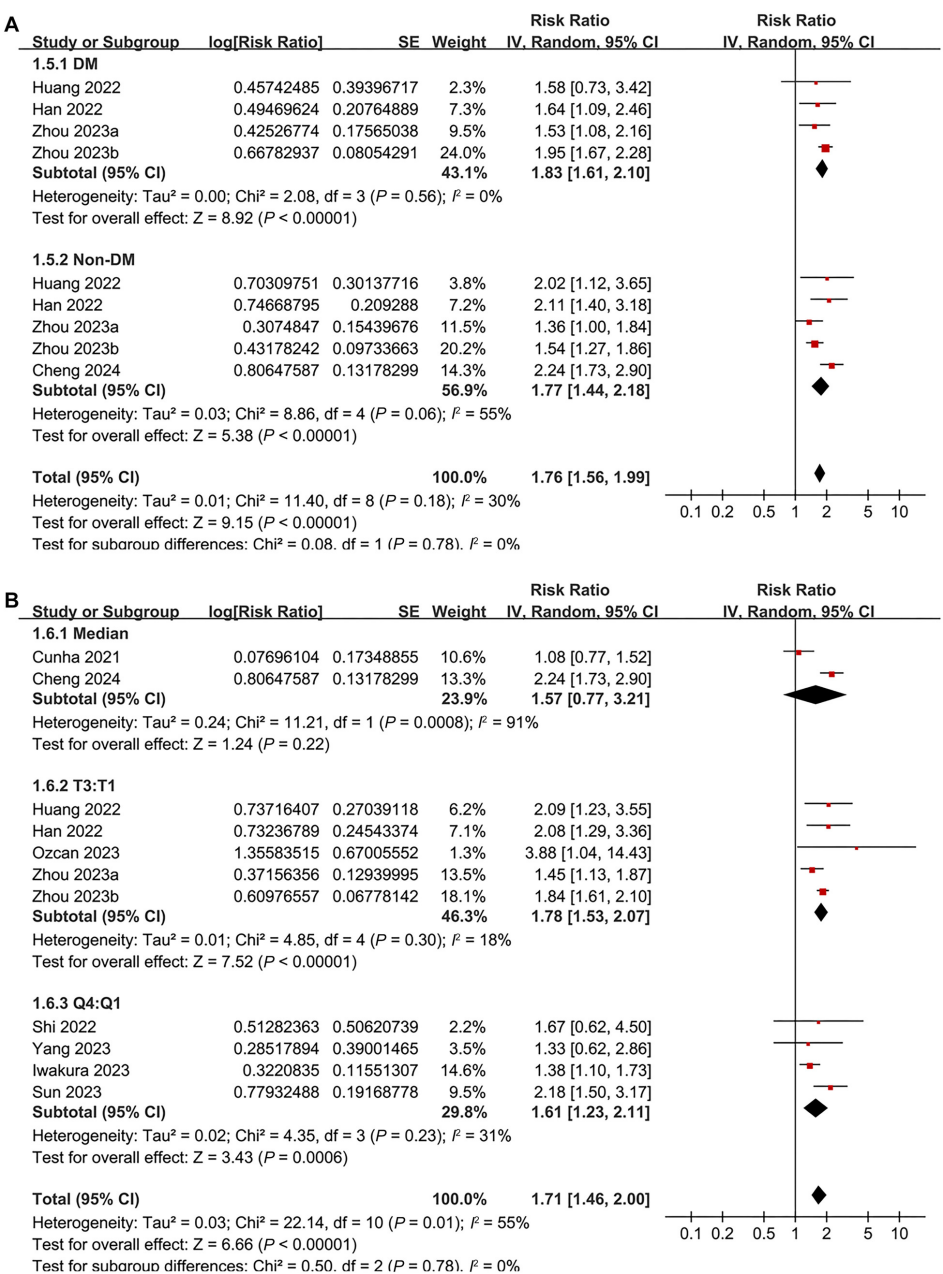
**Forest plots for the subgroup analyses of the association between TyGI and all-cause mortality of patients with HF.** (A) Forest plot for the subgroup analysis based on the diabetic status of the patients; (B) Forest plots for the subgroup analysis based on the methods for determining the TyGI cutoffs. TyGI: Triglyceride-glucose index; HF: Heart failure; DM: Diabetes melitus; T: Tertile; Q: Quartile; CI: Confidence interval.

**Figure 5.**
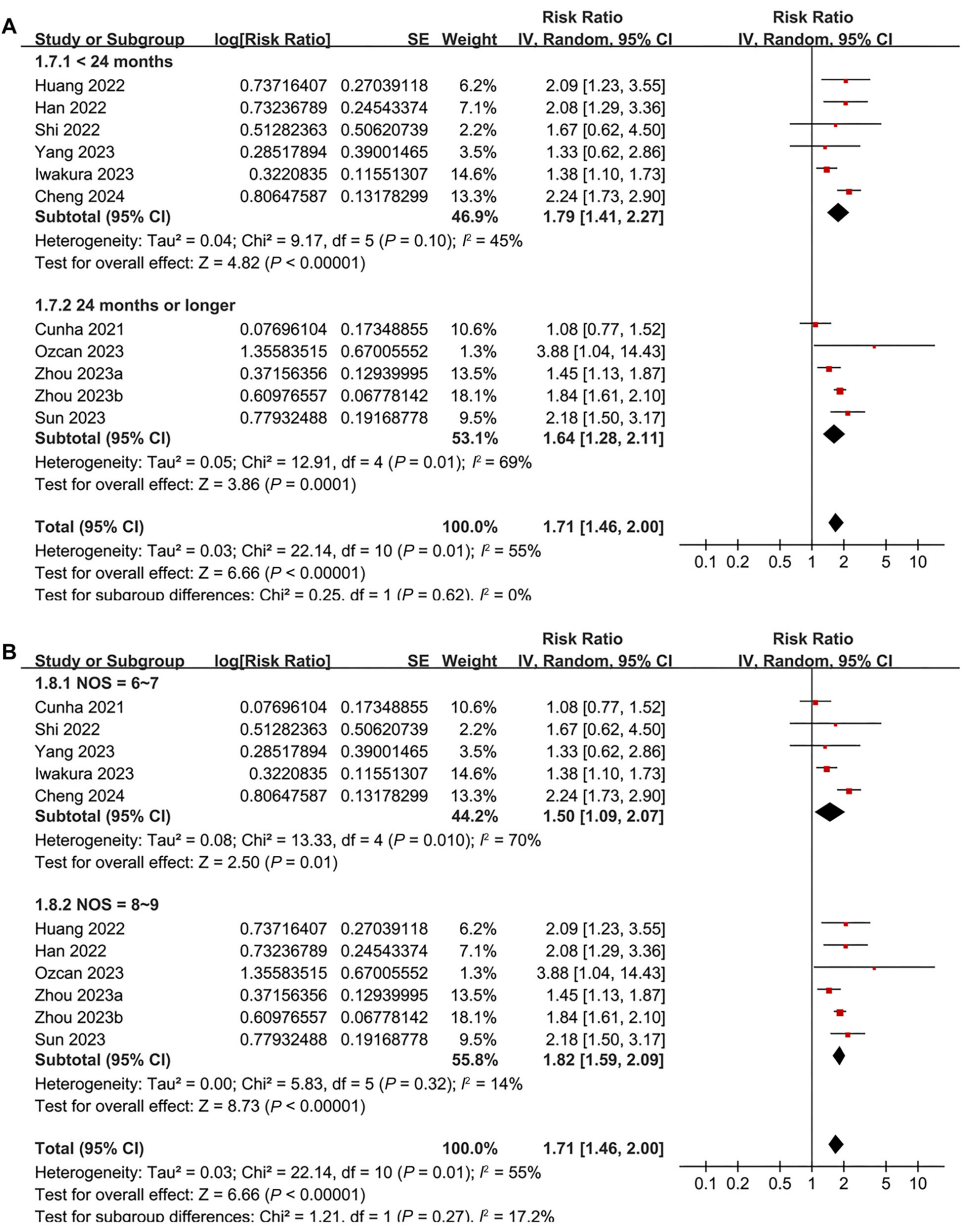
**Forest plots for the subgroup analyses of the association between TyGI and all-cause mortality of patients with HF.** (A) Forest plot for the subgroup analysis based on the follow-up duration; (B) Forest plot for the subgroup analysis according to the study quality scores. TyGI: Triglyceride-glucose index; HF: Heart failure; NOS: Newcastle–Ottawa Scale; CI: Confidence interval.

Pooled results from 11 cohorts [17–27] using a random-effects model indicated that patients with the highest baseline TyGI had a significantly higher risk of all-cause mortality during follow-up compared to those with the lowest TyGI (RR ═ 1.71, 95% CI 1.46–2.00; *P* < 0.001; Figure 2A), with moderate statistical heterogeneity (*I*^2^ ═ 55%). A sensitivity analysis limited to studies with multivariate adjustments for confounding factors showed similar results (RR ═ 1.89, 95% CI 1.67–2.21; *P* < 0.001; Figure 2B), while between-study heterogeneity was notably reduced (*I*^2^ ═ 13%). Subsequent subgroup analyses did not reveal significant differences between patients with AHF and CHF (*P* for subgroup difference ═ 0.33; Figure 3A), or between those with HFrEF and HFpEF (*P* for subgroup difference ═ 0.92; Figure 3B). Additionally, there were no significant differences between diabetic and non-diabetic patients (*P* for subgroup difference ═ 0.78; Figure 4A), between studies with different TyGI cutoffs (*P* for subgroup difference ═ 0.78; Figure 4B), between studies with varying follow-up durations (*P* for subgroup difference ═ 0.62; Figure 5A), or across study quality scores (*P* for subgroup difference ═ 0.27; Figure 5B). Univariate meta-regression analyses further showed that study characteristics such as sample size, mean age, proportion of male participants, proportion of diabetic patients, and follow-up duration did not significantly modify the association between TyGI and all-cause mortality in HF patients (*P* all > 0.05; Table 3).

### Meta-analysis for the association between TyGI and other clinical outcomes

**Figure 6.**
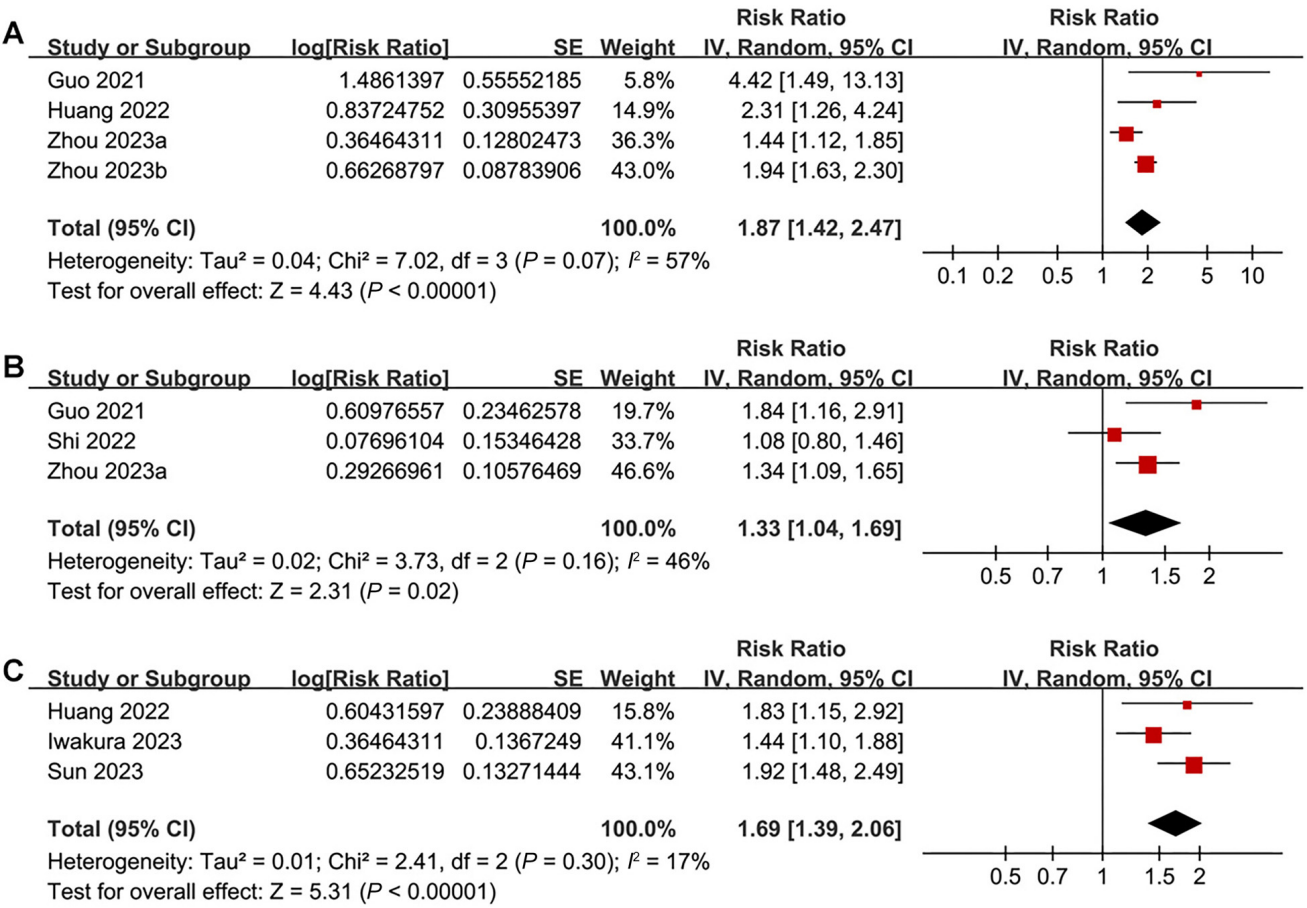
**Forest plots for the meta-analysis of the association between TyGI and the other clinical outcomes in patients with HF.** (A) Forest plot for the association between TyGI and CV death; (B) Forest plot for the association between TyGI and HF rehospitalization; (C) Forest plot for the association between TyGI and MACE. TyGI: Triglyceride-glucose index; HF: Heart failure; CV: Cardiovascular; MACE: Major adverse cardiovascular events; CI: Confidence interval.

Pooled results from four studies [17, 19, 25, 26] indicated that a high baseline TyGI was associated with an increased risk of CV death (RR ═ 1.87, 95% CI 1.42–2.47; *P* < 0.001; *I^2^* ═ 57%; Figure 6A). Similarly, pooled results from three studies [17, 20, 25] showed an association between high TyGI and HF rehospitalization (RR ═ 1.33, 95% CI 1.04–1.69; *P* ═ 0.02; *I*^2^ ═ 46%; Figure 6B). Additionally, results from three studies [19, 21, 23] demonstrated a significant relationship between high TyGI and the incidence of MACE (RR ═ 1.69, 95% CI 1.39–2.06; *P* < 0.001; *I*^2^ ═ 17%; Figure 6C) during follow-up.

### Publication bias

**Figure 7.**
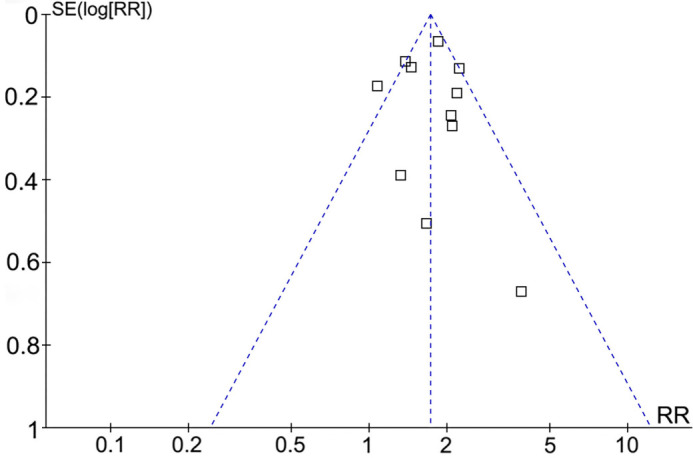
**Funnel plots for the assessment of publication bias in the meta-analysis of the association between TyGI and all-cause mortality in patients with HF.** TyGI: Triglyceride-glucose index; HF: Heart failure.; RR: Risk ratio.

The funnel plots for the meta-analysis of the association between the TyGI and all-cause mortality in HF patients are presented in Figure 7. The symmetrical nature of the funnel plots suggests a low likelihood of publication bias. Additionally, Egger’s regression test confirmed a low risk of publication bias (*P* ═ 0.91). However, publication bias could not be assessed for the meta-analyses of the three secondary outcomes, as they included only three or four studies.

**Table 3:** Univariate meta-regression analysis for the outcome of all-cause mortality

**Variables**	**RR for the association between TyGI and all-cause mortality of HF patients**		
	**Coefficient**	**95% CI**	*P* **values**
Sample size	0.000035	−0.000054 to 0.000123	0.40
Mean age (years)	−0.017	−0.043 to 0.009	0.16
Male participants (%)	0.0055	−0.0107 to 0.0218	0.46
Diabetes (%)	−0.0081	−0.0213 to 0.0051	0.20
Follow-up duration (months)	−0.0048	−0.0140 to 0.0044	0.27

TyGI: Triglyceride-glucose index; RR: Risk ratio; CI: Confidence interval; HF: Heart failure.

## Discussion

This meta-analysis, which included 12 cohort studies, found that patients with HF and a high baseline TyGI had an elevated risk of all-cause mortality during follow-up. Sensitivity analysis confirmed that this association remained significant even after adjusting for potential confounding factors. Subgroup and meta-regression analyses revealed no significant differences in the association between patients with AHF and CHF, between HFrEF and HFpEF, or between diabetic and non-diabetic patients. Study characteristics such as sample size, mean age, proportion of men, methods for determining the TyGI cutoff, follow-up duration, and study quality scores did not significantly affect this association. Furthermore, our analysis suggested that a high baseline TyGI in HF patients was also associated with an increased risk of CV death, HF rehospitalization, and MACE during follow-up. In conclusion, this meta-analysis suggests a potential relationship between high TyGI levels and poor clinical outcomes in HF patients.

This meta-analysis may be the first to comprehensively evaluate the link between baseline TyGI and clinical outcomes in HF patients. It is important to acknowledge several methodological strengths before interpreting the findings. We conducted a thorough search of three widely used electronic databases and identified 12 relevant cohort studies for analysis. By focusing exclusively on cohort studies, the meta-analysis was able to establish a longitudinal relationship between high TyGI and poor prognosis in these patients. Moreover, the sensitivity analysis, which focused on studies with multivariate adjustments for all-cause mortality, showed consistent results and significantly reduced between-study heterogeneity (*I*^2^ from 55% to 13%). These findings support an independent association between high TyGI and increased all-cause mortality risk in HF patients. Additionally, the reduction in *I*^2^ during the sensitivity analysis indicates that univariate analyses may have been a key contributor to heterogeneity. The additional subgroup and meta-regression analyses further strengthened the evidence linking high TyGI to an elevated risk of overall mortality in HF patients. Despite the limited number of studies, our meta-analyses also demonstrated that a high baseline TyGI was associated with an increased risk of CV death, HF rehospitalization, and MACE during follow-up. Collectively, these results suggest that high TyGI could serve as an indicator of unfavorable prognosis for individuals with HF.

Several studies have highlighted the advantages of TyGI as a new indicator of IR. The hyperinsulinemic-euglycemic clamp test is considered the most accurate method for assessing IR, but its complexity and high cost make it impractical for routine clinical use [35]. Alternative indices of IR, such as the homeostatic model assessment of IR (HOMA-IR) and TyGI, are more commonly used in clinical settings. While HOMA-IR is frequently employed [36], TyGI has been proposed as a reliable surrogate marker for IR [37]. Although there is no consensus on the optimal index for IR, a previous study suggested that TyGI correlates more strongly with the hyperglycemic clamp test results than HOMA-IR [38]. The TyGI can be easily calculated using routine biochemical measurements of TG and FPG levels, without the need for insulin assays [9]. Compared to the hyperinsulinemic-euglycemic clamp test, TyGI provides a cost-effective and efficient method for assessing IR, with studies validating its ability to reflect IR severity accurately. An early study demonstrated that the TyG index effectively identifies individuals with IR in diverse populations, including healthy individuals, obese individuals, and patients with diabetes [10]. The TyGI exhibited high sensitivity (96.5%) and specificity (85.0%) compared to the hyperinsulinemic-euglycemic clamp test [10]. Another study in patients with acute ischemic stroke suggested that TyGI might perform better than HOMA-IR [38], further supporting its practical utility as a prognostic indicator.

The association between high TyGI and unfavorable prognosis in HF patients may highlight the significant role of IR in HF progression. In the myocardium, IR and the resulting decrease in cardiac insulin metabolic signaling are increasingly recognized as key contributors to HF development [39, 40]. Factors associated with IR in HF patients include oxidative stress, elevated blood glucose, increased lipid levels, disrupted adipokine/cytokine release, inappropriate activation of the renin-angiotensin II-aldosterone system, and sympathetic nervous system activation, all of which contribute to worsening cardiac function [39, 40]. Additionally, recent research suggests that cardiac IR can directly lead to mitochondrial dysfunction in cardiomyocytes, impairing cardiac metabolic flexibility in HF cases [8, 41]. IR-induced endothelial dysfunction and lipotoxicity may further impair systolic and diastolic function in cardiomyocytes [42], which is also likely to contribute to the association between IR and poor prognosis of HF patients.

There is also growing evidence supporting the potential benefits of metformin, a well-known antidiabetic drug targeting IR, in patients with HF. A previous comprehensive analysis revealed that diabetic patients with HF who used metformin experienced a slight decrease in overall hospitalizations [43]. Another recent meta-analysis indicated that metformin could potentially reduce the risk of all-cause mortality in patients with HFpEF [44]. These findings further support the role of IR as a prognostic factor and potential treatment target for HF patients.

This study has several limitations. Eleven of the included studies were retrospective, which may introduce biases related to selection and recall, potentially affecting the results. Additionally, the meta-analysis protocol was not prospectively registered in PROSPERO. Inconsistency in TyGI cutoff values among the studies contributed to heterogeneity. Further research is required to establish an optimal TyGI cutoff for predicting poor prognosis in HF patients. Although sensitivity analysis focused on studies with multivariate adjustments showed consistent outcomes, unmeasured confounding factors may still influence the results. For instance, none of the included studies reported the methods used to measure FPG and TG, which could affect the association between TyGI and clinical outcomes in HF patients. Additionally, body mass index (BMI), an indicator of obesity status, may influence the association between baseline TyGI and HF prognosis. However, only six studies reported mean BMI values at baseline [17, 19, 20, 23, 25, 26], preventing a thorough assessment of BMI’s impact. Future large-scale prospective studies should address this gap. Lastly, the use of antidiabetic and lipid-lowering medications may also influence the association between baseline TyGI and HF prognosis. Unfortunately, none of the included studies provided stratified data based on the use of these medications, preventing a detailed assessment of their impact. Due to the reliance on observational research, a definitive causal link between high TyGI and poor prognosis in HF patients could not be firmly established.

## Conclusion

The findings of this meta-analysis indicate that patients with HF who have a high baseline TyGI may face an increased risk of adverse clinical outcomes during follow-up compared to those with a low TyGI. Further confirmation through large prospective studies and investigation into the underlying mechanisms is needed. Given the convenience and cost-effectiveness of this parameter, TyGI holds potential as a prognostic marker for HF patients.

## Data Availability

All the data generated during the study are included within the manuscript.
